# CHLA 2026 CONFERENCE PANELS / ABSC CONGRÈS 2026 PANELS

**DOI:** 10.29173/jchla29971

**Published:** 2026-08-03

Lori Ane Oja^1^, Heather Cunningham^2^, Kaitlin Fuller^3^, Annette Flanagin^4^

^1^Health Science Information Consortium, ^2^University of Toronto, ^3^St Francis Xavier University, ^4^JAMA Network

**Topic/Theme:** The integrity of scholarly health information is facing unprecedented disruption. Once unquestioned sources such as PubMed, the CDC, and the NIH are now vulnerable to censorship, politicization, and deliberate misinformation campaigns. These pressures erode trust in authoritative databases and journals, creating ripple effects across research, clinical practice, and public health communication. Public trust in science has been declining for years and has sharply deteriorated in recent times. This panel will examine how health sciences librarians and publishers can respond to these challenges and leverage them as opportunities to demonstrate their essential role. **Panel Overview:** Librarian Responses to Disruption - Critically assess the evolving role of health sciences librarians in countering censorship and misinformation. Positioned at the nexus of teaching, clinical practice support, and research, librarians are equipped to confront these changes and to empower and educate learners, clinicians, and faculty to recognize and resist compromised information. Librarians can leverage disruptions and create strategic opportunities to advocate for evidence integrity and reinforce their value within clinical and research environments. **Monitoring Trusted Sources:** Discuss the need and strategies for monitoring health information sources, such as PubMed and scholarly journals, to detect and interpret shifts in content integrity while developing strategies for communicating these findings to healthcare stakeholders and presenting viable alternative sources. **Publisher Perspective:** Gain insight from a scholarly publisher on efforts to maintain quality and credibility in health information, including editorial safeguards and transparency initiatives. Following brief presentations, a Q&A will allow participants to engage directly with panelists on practical approaches to safeguarding scholarly integrity.

