# Supplementary material for: CHLA 2026 CONFERENCE PANELS / ABSC CONGRÈS 2026 PANELS
Source: J Can Health Libr Assoc. 2026 Aug 3;47(2):88–9. doi: 10.29173/jchla29971 (PMC13590143; doi:10.29173/jchla29971)
Supplement: Related texts

# Abstract: PL2. Librarians as creators of open educational resources for advanced searching

Peter Farrell^1^, Nigèle Langlois^1^, Sandra McKeown^2^, Erica Nekolaichuk^3^

^1^University of Ottawa, ^2^Queen's University, ^3^University of Toronto

**Panel Description:** Health librarians are increasingly authoring their own open educational resources (OERs) to teach advanced literature searching—an essential skill for evidence synthesis. These instructional materials are often created in response to gaps in existing resources and go beyond simple guides by incorporating bilingual content, scaffolded exercises, knowledge checks, and video tutorials. They reflect librarians’ deep expertise and commitment to affordable, open, and high-quality learning materials. This panel will highlight three librarian-led OER projects that exemplify this growing trend. Through brief presentations and a moderated discussion, panelists will share insights into the design, implementation, and impact of their work, and reflect on the broader implications of OER creation as a scholarly and pedagogical practice. **Panelists:** Sandra McKeown (Queen’s University) will present her self-paced module for teaching systematic review search strategies, Erica Nekolaichuk (University of Toronto) will discuss her comprehensive LibGuide for advanced health sciences searching, Peter Farrell (University of Ottawa) will share his and Nigèle Langlois’ bilingual adaptation of Erica’s guide, tailored to the uOttawa context. This panel aims to inspire librarians to see OER creation as a meaningful extension of their teaching and scholarship. Attendees will gain practical ideas for launching their own projects, collaborating within and across institutions, and leveraging funding opportunities to support open instructional design.

# Abstract: PL3. Panel 50th anniversary / Panel 50e anniversaire

Marie-Hélène Nicol^1^, Lee Anne Bourke^2^, David S. Crawford^3^, Kristy Hancock^4^, Martine Gagnon^5^, Janice Thompson^6^

^1^Université de Montréal, ^2^University of Ottawa, ^3^Emeritus Librarian at McGill University, ^4^University of Northern British Columbia, ^5^Université Laval, ^6^William Osler Health System, Brampton Civic Hospital

**Panel Description:** Celebrating the 50th anniversary of the Canadian Health Libraries Association (CHLA), this plenary panel brings together founding leaders, current practitioners, and emerging professionals to reflect on the association’s past, present, and future. Panelists explore the evolution of CHLA, its support for members across diverse settings, the value of service and connection, national advocacy, and the profession’s ongoing adaptation to technological and systemic change. Historical milestones are interwoven with personal stories and reflections from retired members, highlighting the collaborative spirit that has sustained the association for five decades. The panel concludes with a call to action, inviting members to be inspired by this shared history and to actively carry CHLA forward through engagement, leadership, and collective vision as it continues to shape the future of health librarianship in Canada. **Moderator:** Lee Anne Bourke, Associate Librarian, Strategy, University of Ottawa; President, CHLA/ABSC 2014–2015. **Panelists:** David S. Crawford, Founding president of CHLA in 1976 and active member for many years. Emeritus Librarian at McGill University (2002) and CHLA/ABSC Honorary Life Member since 1999. Kristy Hancock, Division of Medical Sciences Librarian, University of Northern British Columbia. Recipient of the CHLA/ABSC Emerging Leader Award 2023. Martine Gagnon, Consulting Librarian, Université Laval. President, CHLA/ABSC 2026. Janice Thompson, Manager, Library and Research Services, William Osler Health System, Brampton Civic Hospital.

**Description du panel :** Célébrant le 50e anniversaire de l'Association des bibliothèques de la santé du Canada (ABSC), cette séance plénière réunit des figures fondatrices, des personnes praticiennes actuelles et des professionnelles et professionnels émergents afin de réfléchir au passé, au présent et à l’avenir de l’association. Les panélistes examinent l’évolution de l'ABSC, son soutien aux membres dans des milieux variés, la valeur de l’engagement et des liens professionnels, le rôle de l’association en matière de représentation nationale, ainsi que l’adaptation continue de la profession aux changements technologiques et systémiques. Des jalons historiques sont entrelacés à des récits personnels et à des témoignages de membres retraités, mettant en lumière l’esprit de collaboration qui soutient l’association depuis cinq décennies. La séance se conclut par un appel à l’action invitant les membres à s’inspirer de cette histoire collective et à contribuer activement à faire progresser l'ABSC par leur engagement, leur leadership et une vision partagée pour l’avenir de la bibliothéconomie de la santé au Canada. **Animatrice :** Lee Anne Bourke, bibliothécaire universitaire associée, Université d’Ottawa, présidente de l'ABSC/CHLA 2014-2015. **Panélistes :** David S. Crawford, fondateur de l'ABSC/CHLA en 1976 et membre actif depuis plusieurs années. Bibliothécaire émérite, Université McGill (2002), membre honoraire à vie de l'ABSC/CHLA depuis 1999. Kristy Hancock, bibliothécaire de la division des sciences médicales, University of Northern British Columbia, récipiendaire du Prix du Flambeau de la relève de l’ABSC/CHLA 2023. Martine Gagnon, bibliothécaire-conseil, Université Laval, présidente de l'ABSC/CHLA 2026. Janice Thompson, responsable des services de bibliothèque et de recherche, William Osler Health System, Brampton Civic Hospital.
